# Response to: methodological considerations for assessing SmO_2_ reproducibility and its applications in sport sciences

**DOI:** 10.1007/s00421-024-05651-8

**Published:** 2024-11-11

**Authors:** Philip Skotzke, Sascha Schwindling, Tim Meyer

**Affiliations:** 1 Institute of Sport and Preventive Medicine, University of Saarland, Campus B8.2, 66123 Saarbrücken, Germany; 2 Olympic Training Center Rhineland-Palatinate/Saarland, Hermann-Neuberger-Straße 2, 66123 Saarbrücken, Germany

We thank Ruiz-Olvera et al. (2025) for their critical appraisal of our study on the reproducibility of muscle oxygenation (SmO_2_) measured with a wearable near-infrared spectroscopy (NIRS) device during cycling (Skotzke et al. 2024). Based on the existing literature, we tried to carefully consider all methodological aspects in designing the study and reporting our results. Still, we appreciate additional feedback and want to provide answers to the comments made in the *Letter to the Editor*. Our response will follow the structure of the letter.

## Assessing and interpreting skinfold thickness

A single trained sport scientist (P. S.) collected all skinfold measurements. All measurements were taken at the same position on the vastus lateralis muscle where the NIRS device was placed during the first visit. It is recognized that a reliability assessment of skinfold measurements by repeating the assessment at all three visits would have been an additional asset and may become part of future standards for NIRS measurements. Consistent with the literature, an investigation at our institute using the same Harpenden skinfold caliper reported coefficients of variation (CV) between 3.8 and 6.1% for two different sport scientists in a combined sample of athletes and patients (unpublished). It is a matter of experience that skinfold measurements become more reliable in leaner individuals. This may mean that the true CV value for our setting was closer to 4% than to 6%.

## Skin tone and phototype assessment

All participants were Caucasians with light skin tone from Europe. Information about ethnicity was not collected. We agree that skin colour should be assessed and reported in an objective way because skin melanin reduces the SmO_2_ signal as more light is absorbed (Barstow 2019). Skin melanin content is a relatively constant trait, and we would argue that there is no basis to assume it affects the *reliability* of SmO_2_ measurements during dynamic movements. This might be different when interindividual comparisons are made or when study duration is long enough to expect changes in skin tone. We recognize that research with subjects of different skin colours would be preferable to conclude the utility of NIRS as a tool in diverse populations. However, such diverse samples (trained cyclists with different skin pigmentation) are hard to acquire.

## Intensity control

Our study aimed to establish the reproducibility of SmO_2_ during steady-state exercise. We believe that such a stable situation is a necessary requirement for using and interpreting NIRS data in real-world training applications. Ruiz-Olvera et al. (2025) propose an investigation into the reproducibility of muscle oxygenation breakpoints. However, this is another type of study. Non-invasive determined exercise thresholds are of some interest, as has been shown by the cited systematic review (Sendra-Pérez et al. 2023). As lactate sampling has been a part of our data collection, it would be possible to compare SmO_2_ breakpoints with lactate-derived thresholds. However, Sendra-Pérez et al. (2023) report five different methods used in the literature for NIRS breakpoint determination, concluding that the most suitable method for a specific sport and testing protocol remains uncertain.

Furthermore, lactate thresholds are based on varying physiological processes, some are a surrogate for the maximal lactate steady-state, others represent the first increase in blood lactate (Faude et al. 2009). The underlying physiology of NIRS breakpoints remains unclear. Thus, comparing NIRS breakpoints with lactate thresholds is therefore associated with some uncertainty and does not follow a distinct research question. Simply calculating correlations between thresholds may be misleading because they are frequently found when they are somehow related to performance. Before test–retest reliability of a surrogate for exercise intensity thresholds is analysed, the validity of the surrogate needs to be established first. Only then should NIRS breakpoints be used in the decision-making process for endurance training.

Once again, we want to thank Ruiz-Olvera et al. (2025) for their constructive comments and hope that answered all questions satisfactorily. A critical discussion of our results and future research directions can help bridge the gap between lab-based physiological testing and the real-world implementation of NIRS technology into training.
